# An emerging consensus in palaeoanthropology: demography was the main factor responsible for the disappearance of Neanderthals

**DOI:** 10.1038/s41598-021-84410-7

**Published:** 2021-03-01

**Authors:** Krist Vaesen, Gerrit L. Dusseldorp, Mark J. Brandt

**Affiliations:** 1 Faculty of Archaeology, Leiden University, Leiden, The Netherlands; 2 School of Innovation Sciences, Eindhoven University of Technology, Eindhoven, The Netherlands; 3 Palaeo-Research Institute, University of Johannesburg, Johannesburg, South Africa; 4 Department of Psychology, Michigan State University, Michigan, USA

**Keywords:** Anthropology, Archaeology

## Abstract

The causes of Neanderthal disappearance about 40,000 years ago remain highly contested. Over a dozen serious hypotheses are currently endorsed to explain this enigmatic event. Given the relatively large number of contending explanations and the relatively large number of participants in the debate, it is unclear how strongly each contender is supported by the research community. What does the community actually believe about the demise of Neanderthals? To address this question, we conducted a survey among practicing palaeo-anthropologists (total number of respondents = 216). It appears that received wisdom is that demography was the principal cause of the demise of Neanderthals. In contrast, there is no received wisdom about the role that environmental factors and competition with modern humans played in the extinction process; the research community is deeply divided about these issues. Finally, we tested the hypothesis that palaeo-anthropologists’ stand in the debate co-varies with their socio-political views and attitudes. We found no evidence for such a correlation.

## Introduction

A long-standing debate in palaeo-anthropology is the demise of Neanderthals approximately 40,000 years ago^1^. Indeed, given their close resemblance to modern humans, and their prolonged success in surviving in Eurasia (about 400,000 years, see^2^, Neanderthals would seem to have all it takes to persist. Still, their phenotype disappeared, even though they left a genetic legacy in the modern human genome. The ultimate disappearance of the Neanderthal phenotype happened concomitantly with migration events by modern humans into the ranges that Neanderthals inhabited^3–7^. Arguably, one of the principal reasons for the continuing and widespread interest in Neanderthal extinction is that it might indirectly tell us something about our own species: the factors contributing to the demise of our sister species might point us to the factors responsible for our own success.

Numerous hypotheses have been advanced to explain the disappearance of Neanderthals. According to a first category of hypotheses, the event was causally related to the migration of modern humans into territories occupied by Neanderthals: resident bands of Neanderthals and incoming bands of modern humans found themselves in competition for the same limited resources. A competitive advantage for modern humans then resulted in the replacement of Neanderthals by the principle of competitive exclusion. This category of hypotheses comprises several variants, distinguishable by the type of competitive advantage they postulate. The inter-specific difference might have been morphological^8,9^, cognitive^10–14^, technological^15–17^, social^18–21^ or economic^22–24^.

A second category pertains to hypotheses that refer to the internal, demographic dynamics of Neanderthal populations. Even in the absence of competition with modern humans, Neanderthal populations might, generally, have been too small to persist in the long run^25–28^. More specifically, their small size and limited interconnectedness would have made them highly susceptible to inbreeding (viz., reduction in fitness of individuals that arise from matings between genetic relatives), Allee effects (reduction in population growth rates due to problems in mate-finding), and stochastic fluctuations (sudden drops in population size due to random fluctuations in births, deaths and sex ratio)^28,29^.

A final, third, category attributes the demise of Neanderthals to environmental factors. These factors include general climatic instability^30–33^, extreme climatic conditions due to volcanic activity^34^, the introduction of pathogens by modern humans into the immunologically naïve Neanderthal population^35–37^.

The current state of the scholarly debate is diffuse. To start, it is difficult to track all contender explanations (Table 1 in the Supplementary Materials is an attempt at doing so) and all their proponents. Further, many hypotheses appear to have more *op*ponents than *pro*ponents. Finally, endorsement of any single explanation may or may not be to the exclusion of other hypotheses. All other things being equal, the fact that an author expresses their support for one explanation does not say much about their support (or lack thereof) for other explanations.

In light of the above, the question arises how much (dis)agreement there is in expert opinion. Answering this question is the first aim of our study. To that effect, we conducted a survey among practicing palaeo-anthropologists. We presented respondents (total number = 216) a list of factors that might have contributed to the disappearance of Neanderthals, and asked them to provide their estimate of the strength of each factor’s contribution. We then assessed the extent to which the research community appeared to have more or less consensus on these issues.

The second aim of our study was to assess correlations between respondents’ views about Neanderthal extinction and a set of social psychological constructs that capture people’s socio-political attitudes. The following considerations motivated us to run such a test. The picture that emerges from at least some of the relevant literature (see, e.g., the exchange between^38–40^) is one of a highly polarized debate, that is divided in ‘tribal’ camps, each defending its own theory.

One possible reason for such clashes is that explanations for Neanderthal disappearance may not be merely reflective of scientific disagreement, but also of disagreement in socio-political attitudes and views. By way of illustration consider the positions of Zilhão^39^ and Wynn et al.^40^. The two parties disagree about the cognitive differences between modern humans and Neanderthals: whereas Wynn and colleagues claim that differences in the cognitive abilities of modern humans and Neanderthals were visible to natural selection, Zilhão believes that the two species were indistinguishable. It could be the case that, as as Zilhão believes, such a difference of opinion has its roots in a difference of opinion about human progress; it could be that, as Zilhão writes, there exists “a persistent, if subconscious influence in academia of Victorian-age ideas of evolution-as-progress and ancient-as-primitive” (p. 52). On Zilhão’s account, claims that Neanderthals are not quite like us betray an outdated form of hierarchical thinking. On Wynn et al.’s account, claims that Neandertals were indistinguishable are rooted in extreme anti-science versions of contemporary social justice theory. However, whether an archaeologist’s scientific views reflect his or her socio-political views or latent psychological motivations remains to be seen.

The idea that socio-political views might bias scientific conclusions is not new. Social psychological studies have found that people are more likely to give the correct answer to a scientific question when it fits with their political worldview^41,42^. In light of the foregoing, we tested if respondents’ views pertaining to social hierarchies and inclusion co-vary with their opinions about Neanderthal disappearance. Is being egalitarian and inclusive towards others associated with views that downplay the causal contribution of competitive differences between modern humans and Neanderthals and that emphasize environmental and demographic factors? In contrast, is being anti-egalitarian and exclusive correlated with views that stress competitive differences and de-emphasize other factors?

To test this, we invited the respondents of our survey also to fill out three validated scales that social and personality psychologists commonly use to assess individuals’ attitudes towards others. The first scale, Social Dominance Orientation (SDO), measures people’s degree of support for group-based hierarchies^43,44^. Second, the Speciesism scale gauges respondents’ inclination to assign different moral worth to members of species other than one’s own^45^. The third is more indirect. We assessed the personality trait Agreeableness, which taps into people’s propensity to express one’s empathy and altruism vis-à-vis others^46^. We tested the extent to which these measures correlated with respondents’ view about Neanderthal disappearance.

There are various reasons for wanting to get a sense of the level of (dis)agreement in the Neanderthal research community (first part of the study)^47^. For one, experts and non-experts (e.g., science journalists) often, implicitly or explicitly, refer to the “received wisdom” among experts. In this sense, it is worthwhile to get clear on what the actual “received wisdom” amounts to when it comes to Neanderthal disappearance. Further, one might take the prevalence of a scientific view as a fallible indication of its truth. IPCC reports on climate change, for example, derive part of their authority from the fact that they represent a consensus view. In a similar vein, a broad consensus among palaeo-anthropologists that, say, inbreeding was a critical factor in the demise of Neanderthals, may be regarded as (fallible) evidence that it indeed was a key factor.

The significance of the second part of our study lies elsewhere. Theoretical work suggests that anthropological theories tend to reflect deeper social, cultural and political commitments (see, e.g.,^48–52^). Such theory-ladenness is to be reckoned with in judgments about objectivity. The results of our study are significant in that specific sense: they allow us to assess the degree to which opinions about Neanderthal disappearance are informed by socio-political presuppositions rather than strictly scientific considerations.

## Methods

The Ethics Committee of the Faculties of Humanities and Archaeology at Leiden University, the Netherlands, reviewed and approved the study protocol. The experiment was performed in accordance with relevant guidelines and regulations. Respondents gave their consent to participating in the study, through a consent form (see Supplementary Materials) that was presented to them prior to filling out the questionnaire (also see Supplementary Materials).

### Questionnaire

We developed an online questionnaire, using the survey software Qualtrics. The questionnaire comprised, in addition to a consent form, three parts. The first part asked respondents to give their opinion on the likely causes of Neanderthal disappearance. Respondents were requested to estimate the strength of the causal contribution of a set of *demographic factors* (viz., inbreeding, stochasticity, population size, Allee effects, other demographic factors), a set of *environmental factors* (viz., climatic factors, epidemics, other environmental factors), and a set of *factors pertaining to competitive interaction between modern humans and Neanderthals* (viz., cognitive advantages of modern humans, technological advantages of modern humans, social advantages of modern humans, economic advantages of modern humans, morphological advantages of modern humans, other advantages of modern humans, advantages of Neanderthals over modern humans). These three types of factors were randomly presented to respondents. Additionally, respondents were requested to indicate whether they had, over the last 5 years, endorsed those factors in their own scholarly writing, and to estimate their general uncertainty about the explanations they endorsed.

The second part consisted of three validated scales: participants completed a measure of Social Dominance Orientation^43,44^, Speciesism^45^, and Agreeableness^46,53^.

Social dominance orientation is a well validated political value measure^43^. People with higher scores endorse anti-egalitarian values and the dominance of powerful groups, and people with lower scores endorse egalitarian values and reject the dominance of powerful groups. In prior work, higher values on this scale have been related to political conservatism, support for right-wing political parties, and prejudice towards disadvantaged and marginalized groups (e.g.,^54^). We selected a balanced set of 4 items from the most recently validated short version of the scale^43^. The short version of the scale typically includes 8 items,however, we wished to keep the survey as short as possible given the busy lives of our participants.

Speciesism is a well-validated measure of people’s beliefs regarding the superiority of humans compared to other animals^45^. People who score high on this measure see humans as distinct and morally valuable compared to other animals, whereas people who score low on this measure see humans and animals as more similar. In prior work, higher values on this scale have been related to less positive views of animals, less empathetic concern, and higher levels of social dominance^45,55^. We used 5 of the 6 items of the validated scale. We removed one item about the legal rights of Chimpanzees because feedback from our colleagues suggested that this item would be viewed as strange among our participant sample.

Agreeableness is a personality trait (one of the Big 5 personality traits^46^) representing people’s tendency to get along well with other people. This measure does not directly measure attitudes about inclusion and exclusion, but instead taps into these ideas more indirectly. In prior work, agreeableness has appeared to be associated with lower levels of prejudice towards social groups, less hostility, less entitlement, and lower levels of social dominance^43,53,56^. There are multiple measures of this construct. We chose to use the brief version from the “mini-International Personality Item Pool”^53^.

The third part comprised a set of questions concerning the demographics of the respondents (viz., age, gender, current position, familiarity with Neanderthal archaeology, field of specialization, nationality, country of residence, country of PhD, time since PhD).

Finally, in order to conceal the aim of the study (its second aim in particular), the questionnaire contained two distraction questions.

### Participants

Participants were recruited by direct recruitment and recruitment through social media. As to the former, we collected the email addresses of experts, based on their written scientific outputs. Experts were defined as researchers who had served as a corresponding author for an article that: (1) was published, during the period 01.01.2014–01.04.2020, in *Science*, *Nature*, *Proceedings of the National Academy of Sciences*, *PLoS ONE*, *Evolutionary Anthropology*, *Current Anthropology*, *Journal of Human Evolution*, *Journal of Archaeological Science*, *Quaternary International*, *Bulletin de la Société Préhistorique Française*, or *Archäologisches Korrespondenzblatt*; (2) mentioned the terms “Neanderthal” or “Neandertal” in the body of the text (excluding the bibliography). For the general science journals *Science*, *Nature*, *Proceedings of the National Academy of Sciences*, and *PLoS ONE*, we added an extra filter and retained only corresponding authors that were affiliated to an anthropological or archaeological institute. In the resulting list, we removed all authors that had some professional affiliation to the Faculty of Archaeology at Leiden University (the home institute of Vaesen and Dusseldorp). The 622 remaining researchers were invited by email to fill out the online questionnaire. We sent a reminder email seven days after the first invitation, and another seven days later. Twenty of our invitees in fact never received our invitation; their server sent us a “unable to deliver” notification. Given this, and given a total of 176 responses, the response rate of our direct recruitment is 29.2%.

A second batch of respondents (N = 65) was recruited via Twitter (Dusseldorp’s account).

To make it attractive for potential respondents to complete our survey, we organized a lottery, which awarded a $500 gift (ancient DNA test or Amazon voucher) to one of the respondents.

### Data analysis

We used the statistical software package R for our data analysis. Our analyses included repeated ANOVA, paired t-tests, correlations, and paired Pitt–Morgan tests. Details about our analyses are integrated in the Results section.

## Results

### Descriptive statistics

A total of 295 respondents opened the questionnaire. Of these, 216 respondents completed at least one of the questions of the first part of the questionnaire (pertaining to the factors for disappearance) and were included in subsequent analyses (the 79 people who opened the questionnaire but did not answer any questions about the factors for disappearance also did not complete any other items). Participants predominantly (81%) had reached the questionnaire via our direct recruitment procedure. Fifty-five percent of the respondents identified as male, 30% as female, while the remaining 15% didn’t wish to answer or had missing data for this question. Our recruitment procedure appears to have, as intended, yielded an expert sample: 87% of the respondents were engaged in academic research (2% reported other types of positions that were not obviously research positions, and 11% did not answer this question). The average score for respondents’ familiarity with Neanderthal archaeology was 4.2 (out of 5), 79% of the respondents worked in the subfields “Palaeoanthropology” or “Archaeology” (rather than “Biochemistry or Palaeogenetics”), and 75% were specialized in the Lower and Middle Palaeolithic, Lower and Middle Stone Age, Upper Palaeolithic, Later Stone Age, or some combination.

Table 1 represents the mean scores and standard deviations (SDs) on the individual items pertaining to the likely causes of Neanderthal disappearance, as well as the average scores and SDs of the composite measures Demography (DEM), Competition (COMP), and Environment (ENV). The percentage of times respondents indicated “don’t know” for each of the individual items is also included. These composite measures were calculated by averaging the scores of the individual items relating to, respectively, demography, competition and environment (“don’t know” responses were treated as missing data). Support for demographic factors was moderately correlated with support for environmental factors (r(206) = 0.29, *p* < 0.001), but not with support for competition factors (r(209) = 0.12, *p* = 0.09). Support for environmental factors and support for competitive factors were also weakly correlated (r(205) = 0.19, *p* = 0.006). Table 2 in the Supplementary Materials lists the demographic, competitive and environmental factors that respondents entered in the question box “Other factors”, i.e., causal factors other than those listed by us but found relevant by some of our respondents. Virtually all such “Other factors” were either demographic, competitive or environmental. Only two respondents mentioned a factor that is not demographic, competitive or environmental. More in particular, they indicated that the disappearance of the Neanderthal phenotype was (partially) due to Neanderthal populations being (genetically) merged into modern human populations. Three respondents, finally, mentioned an “Other factor” that was hard to interpret and classify. For completeness sake, Table 3 in the Supplementary Materials contains the competitive advantages of Neanderthals over modern humans that respondents entered in the relevant box. Although such advantages cannot explain the demise of Neanderthals, they are interesting in their own right.

**Table 1:** Mean, minimum and maximum scores, and standard deviations (SD) for the items in the questionnaire, as well the percentage of respondents who indicated “Don’t know”.

	Mean	SD	Minimum	Maximum	% Don't Know
**Causal factors**					
*Demographic composite (DEM)*	3.41	1.13	0	6	
Allee effects	2.74	1.60	0	6	17.45
Inbreeding	3.12	1.59	0	6	4.67
Population size	4.44	1.36	0	6	2.78
Stochasticity	3.03	1.54	0	6	7.51
*Competitive composite (COMP)*	2.40	1.41	0	6	
Cognitive advantage(s)	2.19	1.81	0	6	4.19
Economic advantage(s)	2.63	1.80	0	6	13.15
Morphological advantage(s)	1.51	1.56	0	6	4.67
Social advantage(s)	3.19	1.83	0	6	7.48
Technological advantage(s)	2.60	1.88	0	6	1.40
*Environmental composite (ENV)*	3.08	1.54	0	6	
Climatic factors	3.20	1.72	0	6	2.34
Epidemics	2.76	1.72	0	6	30.19
**Social attitude scales**					
Social dominance orientation	2.01	0.99	1	7	
Speciesism	2.55	1.30	1	7	
Agreeableness	4.19	0.69	1	5	

Table 1 also summarizes the scores on the composite measures of the Social Dominance Orientation (SDO), Speciesism (SPEC) and Agreeableness (AGREE) scales. Composite scores were obtained by averaging the scores of the individual items. Scores for these scales are in line with expectations from past work. For example, samples of U.S. Americans typically have social dominance orientation scores that are approximately 2.5 out of 7^43^. Our sample has lower scores, consistent with the tendency for academics to be more left-wing than the general population^57^, and consistent with a recent survey among anthropologists^58^. Samples of U.S. Americans also tend to score higher on speciesism (e.g., M = 3.46 out of 7 in^45^ than our sample. For agreeableness, U.S. American student samples tend to score similarly to our own sample (Ms between 4.01 and 4.16 in^53^). The three scales were also intercorrelated as expected^43,45^. People who scored higher on SDO also scored higher on SPEC (r(185) = 0.31, *p* < 0.001) and lower on AGREE (r(188) = − 0.41, *p* < 0.001). SPEC and AGREE were also negatively correlated (r(184) = − 0.33, *p* < 0.001).

Responses pertaining to causal factors and responses pertaining to socio-political views did not significantly vary according to respondents’ demographic characteristics (gender, age, etc.).

### Ranking of causal factors

The scores on the composite measures DEM, COMP, ENV were statistically different from one another (F(2, 410) = 35.80, *p* < 0.001). We found that respondents thought demographic factors (M = 3.41, SD = 1.13) were larger contributors than environmental factors (M = 3.08, SD = 1.54, t(207) = 2.87, *p* = 0.005), which were thought to be larger contributors than competitive factors (M = 2.40, SD = 1.41, t(206) = 4.95, *p* < 0.001). In short, the ranking of the scores was COMP < ENV < DEM. Spelled out in full, respondents thought that the causal contribution of demographic factors to the demise of Neanderthals is stronger than the contribution of environmental factors, and that the contribution of the latter is stronger than the contribution of competitive factors.

Table 2 ranks the support for the individual causal factors, based on their mean score. Population size has the most support (mean = 4.44), is the only individual factor with a mean score higher than a 4 (the scale ranges from 0 to 6), and is significantly higher than the second ranked factor (paired t(204) = 8.55, *p* < 0.001). Morphological advantage(s) has the least support (mean = 1.51), is the only individual factor with a mean score lower than a 2, and is significantly lower than the second lowest factor (paired t(196) = 5.03, *p* < 0.001). The remaining factors fall within 1 point of the midpoint of the scale.

**Table 2:** Ranking of causal factors.

Causal factors	Mean	count	Statistically different?
Population size	4.44	109	***
Climatic factors	3.20	87	ns
Social advantage(s)	3.19	77	ns
Inbreeding	3.12	49	ns
Stochasticity	3.03	34	ns
Epidemics	2.76	19	ns
Allee effects	2.74	26	ns
Economic advantage(s)	2.63	46	ns
Technological advantage(s)	2.60	63	***
Cognitive advantage(s)	2.19	39	***
Morphological advantage(s)	1.51	25	

Ranking based on mean scores. “Count” refers to the number of times respondents indicated they had endorsed the factor in their own work. The “Statistically different?” column tests if the mean of the higher ranked factor is significantly different from the next lowest ranked factor using paired t-tests, where ***p < 0.001 and ns *p* > 0.05.

Table 2 (third column) also shows, for each causal factor, the number of times that respondents indicated they had endorsed the factor in their own work. A ranking that would be based on these counts would be very similar to the ranking obtained based on the mean scores (as in the Table). A notable exception are the competitive factors “Economic advantage”, “Technological advantage” and “Cognitive advantage”, factors that would rank higher if the ranking were based on endorsement counts alone. Note, however, that these factors had low consensus scores (see their SD’s in Table 1, and see below); these low consensus scores probably explain the discrepancy between the factors’ relatively low means and their relatively high endorsement counts.

### Level of disagreement

There was variation in support across all of the factors. A series of paired Pitt-Morgan tests showed that there was more variation in both environmental factors (t(206) = 4.57, *p* < 0.001) and competitive factors (t(209) = 3.17, *p* = 0.002) than demographic factors, suggesting that there was less consensus for these factors. Variance in environmental and competitive factors did not differ from each other (t(205) = 1.32, *p* = 0.19).

Regarding individual items, the SDs are in Table 1. As expected from the composite comparisons, the highest SDs are for items that are part of the competition and environment composites. The lowest SDs are all for items that are part of the demographic composite.

### Correlations between explanatory factors and socio-political attitudes

We tested the correlations between, on one hand, SDO, SPEC, and AGREE and, on the other, the composite measures DEM, COMP, ENV. These correlations are in Table 3. These tests yielded only one correlation, namely a positive correlation between agreeableness and demographic factors; however, this correlation did not survive multiple corrections (corrected *p* = 0.15). We conducted the same analyses, but also controlled for gender, time since PhD, and country where the participant worked. These partial correlations are also in Table 3. All of the conclusions are identical, with one exception, more specifically, a small negative correlation between AGREE and COMP. The same correlational analyses were conducted for the individual items. Two of the 33 correlations were significant (SDO-Population size r = − 0.17, *p* = 0.02; SPEC-technological advantages r = 0.15, *p* = 0.04), and neither of these correlations survived multiple corrections. In short, social views do not appear to be meaningfully correlated with researchers’ positions on this debate.

**Table 3:** Correlation (r) and partial correlation (pr) tests between social attitudes and composite causal factors DEM, ENV, COMP.

Social views	Composite factor	*r*	*p*	*pr*	*p*
Social dominance orientation (SDO)	DEM	− 0.07	0.36	− 0.07	0.37
Social dominance orientation (SDO)	ENV	− 0.03	0.74	− 0.03	0.75
Social dominance orientation (SDO)	COMP	0.04	0.58	0.08	0.31
Speciesism (SPEC)	DEM	− 0.01	0.94	0.04	0.65
Speciesism (SPEC)	ENV	− 0.02	0.77	0.02	0.77
Speciesism (SPEC)	COMP	0.11	0.13	0.13	0.10
Agreeableness (AGREE)	DEM	0.17	0.02	0.18	0.02
Agreeableness (AGREE)	ENV	− 0.02	0.76	− 0.05	0.50
Agreeableness (AGREE)	COMP	− 0.07	0.32	− 0.16	0.04

## Conclusion and discussion

Our results are striking in several respects. To start, demographic factors are generally considered to be the most salient in accounting for the disappearance of Neanderthals. Our expert sample thought the strength of the causal contribution of demography to be larger than the strength of the contribution of either environmental of competitive factors: the DEM composite scored significantly higher than the ENV and COMP composites; the individual factor with the highest score appeared to be a demographic one, viz. “Population size”; and two other demographic factors made it to the top five of the ranking (“Inbreeding” and “Stochasticity”). Environmental factors are the runner-up: ENV scored significantly higher than COMP, and climatic factors ranked second in the ranking of individual factors (i.e., before any competitive factor).

Also in terms of consensus, demography has the highest scores: both the DEM composite and, at the individual factor level, “Population size” exhibited the lowest variance. We found no significant difference in the level of disagreement in the ENV and COMP composites.

So, what is the received wisdom among practicing palaeo-anthropologists? The high consensus scores for demography suggest that the current received wisdom pertains to demography. More specifically, according to received wisdom, the disappearance of Neanderthals was *primarily* driven by demographic factors. This is surprising, given that, according to a recent review of the literature^59^, virtually all archaeological studies of the Neanderthal-*H. sapiens* transition that have explicitly evoked a causative role for such factors were published in the last decade (for a similar point, see^25^) most of these studies even came out just during the past 5 years. In fact, demography has only very recently been shown to be sufficient to account for the demise of Neanderthals^26,28^.

Because our survey is the first in its kind, we are not in a position to track the received wisdom over time, and to assess which explanation best accounts for our results. The results might indicate a recent convergence of opinion concerning the causative role of demographic factors, and this after a long period during which, according to French^25^, competitive factors received most attention. Alternatively, demography might have long been popular among palaeo-anthropologists, but have, not until recently, become explicitly endorsed in scholarly publications.

Noteworthy, too, is that there simply is *no* received wisdom regarding other possible causal factors. The disagreement over the supposed cognitive, technological, social, economic advantages of modern humans over Neanderthals that characterizes the aforementioned exchange between Villa and Roebroeks^38^, Zilhão^39^, and Wynn et al.^40^, does not seem to be just a disagreement among a couple of individuals,rather, these issues appear to divide the entire research community. This finding is at odds with Breyl’s literature review^59^. According to Breyl, the received wisdom among palaeo-anthropologists is that Neanderthals were cognitively and technologically comparable to modern humans. This divergence of results might be due to the fact that Breyl’s review, given its non-systematic nature, is incomplete—and, indeed, Breyl seems to miss at least some relevant papers, including Wynn and Coolidge^14^, Pearce et al. and Collard et al.^16^. Alternatively, published statements, as reviewed by Breyl, might not accurately reflect what the broader research community thinks about the issue.

We did not find any correlation between respondents’ socio-political views and their views about competitive causal factors (or any other causal factor, for that matter). Moreover, the level of disagreement about competitive factors was, at the statistical significance level, indistinguishable from the level of disagreement about environmental factors. This runs counter to the hypothesis that the strong disagreement about competitive factors reflects a deeper disagreement; more specifically, a disagreement about socio-political attitudes and views (following Zilhão’s suggestion^39^ and conforming to the hypothesis of the second part of our study).

Our finding that socio-political presuppositions, or at least presuppositions along the traditional left–right political spectrum, do not determine archaeologists’ scientific views is interesting also in the sense of refuting the complaint that anthropology is nothing but politics disguised as science^58^. As far as our results go, disagreements about the demise of Neanderthals are *scientific* disagreements; arguably, they primarily result from the general difficulties in inferring behavioral and other traits from a low-resolution and poorly preserved archaeological record.

As to the limitations of our study, our achieved response rate (29.2%), although high in comparison to studies similar to ours^60,61^ and resulting in a solid statistical power (80% to detect an effect size of *r* = 0.19), does not allow us to fully rule out sampling biases. We cannot fully rule out, for instance, that invitees who endorse anti-egalitarian and exclusive views were, given the generally liberal and left-wing climate among academics, reluctant to fill out our survey. A second concern is that our study relies on self-reporting. Particularly relevant in the context of our study are social desirability biases in self-reports. Indeed, it is not unreasonable to think that our questions about respondents’ socio-political views prompted socially desirable answers. In our study, this would mean that respondents adjusted their answers so that they aligned with, among academics, dominant, liberal and left-wing ideologies. If this were the case, even if a correlation between opinions about Neanderthals and socio-political views existed, our methodology would not allow us to establish it. There are two reasons for not worrying too much about social desirability biases, though. First, it was made clear in advance to respondents that their answers would be anonymous; such anonymity makes it more inviting for respondents to be frank about their socio-political commitments. And, second, the socio-political views of our sample are in line with the views of academics from other disciplines^57^ and with the leftist orientation of anthropologists in particular (Horowitz^58^, see also above, Results > Descriptive statistics).

These possible limitations are impossible to fully avoid. But despite them, our survey seems a good way of getting at the received wisdom of the research community: it is fully anonymous, and it is much broader in scope than the review of Breyl^59^. Whereas Breyl restricted himself to published statements (and did so in a non-systematic way, see above), our method allowed us to gauge the opinions of a much larger, and hence more representative pool of experts. In any case, our survey yields an, even if fallible, answer to the research questions we set out to resolve. To reiterate, according to received wisdom, demography was the principal driver of Neanderthal disappearance,there is no received wisdom pertaining to the causative role of environmental and competitive factors; and experts’ views about the disappearance of Neanderthals do not appear to be driven by these experts’ socio-political attitudes.

Our study has a clear time-stamp: it has revealed the research community’s views *anno* 2020. Accordingly, it may serve as a benchmark for future studies about received wisdom. We plan, in fact, to regularly (with time intervals of 5 years) launch surveys of the kind described in the present paper. Doing this will allow us to track any developments in what researchers believe about the still enigmatic disappearance of our sister species.

## Supplementary Information

Supplementary Information

## Data Availability

See Supplementary Materials attached to this submission. For the experimental data, see https://osf.io/z9rd3/?view_only=ac4fe309454447bab8337ab2e8664f54.
