# Author Correction: An emerging consensus in palaeoanthropology: demography was the main factor responsible for the disappearance of Neanderthals

**DOI:** 10.1038/s41598-021-88189-5

**Published:** 2021-04-13

**Authors:** Krist Vaesen, Gerrit L. Dusseldorp, Mark J. Brandt

**Affiliations:** 1 Faculty of Archaeology, Leiden University, Leiden, The Netherlands; 2 School of Innovation Sciences, Eindhoven University of Technology, Eindhoven, The Netherlands; 3 Palaeo-Research Institute, University of Johannesburg, Johannesburg, South Africa; 4 Department of Psychology, Michigan State University, Michigan, USA

Correction to: *Scientific Reports* https://doi.org/10.1038/s41598-021-84410-7, published online 01 March 2021

The original version of this Article contained an error in discussing the positions of Zilhão, and Wynn and colleagues. The text in the Introduction section,

“By way of illustration, consider the exchange between Zilhão^39^ and Wynn et al.^40^. The two parties profoundly disagree about the cognitive differences between modern humans and Neanderthals: whereas Wynn and colleagues claim that the cognitive sophistication of modern humans markedly exceeded that of Neanderthals, Zilhão believes that the two species were indistinguishable. It could (!) be the case that, as Zilhão suggests, this difference of opinion has its roots in a difference of opinion about human progress; it could (!) be that, as Zilhão writes, Wynn et al. are afflicted by “a persistent, if subconscious influence … of Victorian-age ideas of evolution-as-progress and ancient-as-primitive” (p. 52). On Zilhão’s account, claims that Neanderthals are not quite like us betray an outdated form of hierarchical thinking.”

now reads:

“By way of illustration consider the positions of Zilhão^39^ and Wynn et al.^40^. The two parties disagree about the cognitive differences between modern humans and Neanderthals: whereas Wynn and colleagues claim that differences in the cognitive abilities of modern humans and Neanderthals were visible to natural selection, Zilhão believes that the two species were indistinguishable. It could be the case that, as Zilhão believes, such a difference of opinion has its roots in a difference of opinion about human progress; it could be that, as Zilhão writes, there exists “a persistent, if subconscious influence in academia of Victorian-age ideas of evolution-as-progress and ancient-as-primitive” (p. 52). On Zilhão’s account, claims that Neanderthals are not quite like us betray an outdated form of hierarchical thinking. On Wynn et al.’s account, claims that Neandertals were indistinguishable are rooted in extreme anti-science versions of contemporary social justice theory. However, whether an archaeologist’s scientific views reflect his or her socio-political views or latent psychological motivations remains to be seen.”

This has now been corrected in the PDF and HTML versions of the Article.

