# Supplementary material for: An emerging consensus in palaeoanthropology: demography was the main factor responsible for the disappearance of Neanderthals
Source: Sci Rep. 2021 Mar 1;11:4925. doi: 10.1038/s41598-021-84410-7 (PMC7921565; doi:10.1038/s41598-021-84410-7)
Supplement: Supplementary file 1 — Supplementary Information [file 41598_2021_84410_MOESM1_ESM.pdf]

SUPPLEMENTARY MATERIALS

An emerging consensus in palaeoanthropology: demography was the main factor responsible for the disappearance of Neanderthals

Krist Vaesen[1,2], Gerrit L. Dusseldorp[1,3], Mark J. Brandt[4]

[1] Faculty of Archaeology, Leiden University, The Netherlands

[2] School of Innovation Sciences, Eindhoven University of Technology, The Netherlands

[3] Palaeo-Research Institute, University of Johannesburg, South Africa

[4] Department of Psychology, Michigan State University, U.S.

Corresponding author: k.vaesen@tue.nl

ORCIDs

K Vaesen 0000-0002-7496-7463

G Dusseldorp 0000-0001-6147-710X

M Brandt 0000-0002-7185-7031

SI Table 1: Overview of recently proposed explanations for Neanderthal disappearance

Factor	Sub-factor	Exemplars
Morphology	Neanderthals' shorter lower limbs lead to increased cost of locomotion compared to AHMs; potentially leads to competitive disadvantage in mobility organisation and exploitation of spatially dispersed resources	Steudel-Numbers 2004
	Neanderthal calcaneus points to foot structure less suited to endurance running than AMHs; potentially leads to competitive disadvantage in mobility organisation and hunting strategies	Raichlen et al. 2011
	Neanderthals were adapted to "power locomotion" in rugged, bushy terrain. They were at a disadvantage in increasingly open landscapes.	Stewart et al. 2019; Bastir et al. 2020
	High energetic requirements led to smaller population sizes than AMHs in similar circumstances; also led to longer inter-birth intervals, meaning slower recovery from population crashes and slower re-colonization of depopulated areas.	Sorensen and Leonard 2001; Froehle and Churchill 2009
Cognition	Lower working memory in Neanderthals leads to less frequent innovation, less ability in cost-benefit analysis	Wynn and Coolidge 2004
	Neanderthals have sophisticated domain-specific intelligence, but domains not well-integrated, hence	Mithen 1996; 1999
	Limited capacities for symbolic communication including language compared to AMHs	Mellars 2004
	Limited linguistic capabilities compared to AMHs	Bolhuis et al. 2014; Berwick and Chomsky 2017
	Neanderthals had larger orbits, adaptation to lower light levels. In conjunction larger part of the neocortex was taken up by visual system leaving less processing capacity for social cognition putting them at disadvantage compared to AMHs	Pearce et al. 2013
	AMHs increased spatial cognition and extensive networks aids rapid dispersal and colonization and relatively greater evolutionary success of modern humans over Neanderthals	Burke 2012
	Brain anatomy: relatively smaller cerebellum in Neanderthals led them to have less information processing capacity than AMHs	Kochiyama et al. 2018
Environment	Climatic instability leading to depopulation of regions by Neanderthals clears the way for AMH colonization before Neanderthal populations rebounded	Müller et al. 2011
	Modelling of ecological niches of Neanderthals and AMHs suggests the area suitable for both species declined in late MIS 3, but area suitable for Neanderthals was more fragmented making their populations more vulnerable	Finlayson and Carrión 2007; Melchionna et al. 2018
	Succession of cold, arid events led to Neanderthal depopulation of areas and AMH recolonisation	Staubwassser et al. 2018
	Neanderthals exposed to new pathogens to which AMHs had already built up resistance.	Houldcroft and Underdown 2016; Greenbaum et al. 2019
	Volcanic eruptions in Italy and Caucasus leading to environmental deterioration may have led to Neanderthal local extinctions and AMH recolonization	Golovanova et al. 2010

**Technology**	Neanderthals likely did not use tailored cold-weather clothing, in contrast to AMH giving the latter competitive advantage	Collard et al. 2016
	Neanderthals did not produce projectile weapons while AMH did	Shea and Sisk 2010
	Neanderthals were unable to make fire at will, only “harvested” naturally occurring fire	Dibble et al. 2018
	Different spatial structure of sites, reflecting different social organisation; no dwelling constructions may be disadvantageous in the cold	Kolen 1999; Chu 2009
	Neanderthal prepared core knapping strategies led to less useful debitage products than AMH blade and bladelet production from same quantity of raw material	Bar-Yosef & Kuhn 1999; Muller and Shipton 2016
**Economy**	Less exploitation of small, fast-moving prey through e.g. trapping by Neanderthals gives competitive advantage to AMH who have more biomass at their disposal. Due to high reproductive rate, small mammals and birds also more resilient to over-exploitation and rebound faster from population crashes.	Stiner and Kuhn 2006
	Less exploitation of aquatic resources than AMH gives competitive advantage to the latter	Richards et al. 2001<br>Marean 2014
	Less effective resource exploitation than AMH gives competitive advantage to the latter	Timmermann 2020
	Less effective resource exploitation than AMH gives competitiOutcompeted by AMH groups hunting with domestic dogs, which greatly increases hunting activitiesve advantage to the latter	Shipman 2015
**Social**	Less trade between groups leads to less efficient exploitation and less options to buffer scarcity	Horan et al. 2005
	Inter-group alliances limited compared to AMH, as demonstrated by absence/rarity of evidence for long-distance exchange of materials, who were thus better to deal with adverse conditions	Gamble 1999
**Demography**	Small-group size (and limited intergroup contact due to low population density), lead to dearth of suitable mating partners resulting in frequent episodes of inbreeding and allee effect, depressing fitness	Vaesen et al. 2019; Sikora et al. 2017
	Numerical supremacy: 10-fold larger population density of AMH compared to Neanderthals, important competitive factor	Mellars and French 2011
	Higher mortality/lower life expectancy than AMH	Ramirez-Rossi and Bermudez de Castro 2004
	Higher energetic needs compared to AMH led to longer inter-birth interval, lower offspring survivorship among Neanderthals	Froehle and Churchill 2009

References

Bar-Yosef, O. & Kuhn, S. L. The big deal about blades: laminar technologies and human evolution. Am. Anthropol. 101(2), 322–338 (1999).

Bastir, M. et al. Rib cage anatomy in Homo erectus suggests a recent evolutionary origin of modern human body shape. Nat. Ecol. Evol. 4(9), 1178–1187 (2020).

Bruner, E. et al. Cognitive archeology, body cognition, and hand–tool interaction. Prog. Brain Res. 238, 325–345 (2018).

Burke, A. Spatial abilities, cognition and the pattern of Neanderthal and modern human dispersals. Quat. Int. 247, 230–235 (2012).

Chu, W. A functional approach to Paleolithic open-air habitation structures. World Archaeol. 41(3), 348–362 (2009).

Dibble, H. L. et al. Were Western European Neanderthals able to make fire?. J. Palaeolithic Archaeol. 1, 54–79 (2018).

Eren, M. I., Greenspan, A. & Sampson, C. G. Are Upper Palaeolithic blade cores more productive than Middle Palaeolithic discoidal cores? A replication experiment. J. Hum. Evol. 55(6), 952–961 (2008).

Finlayson, C. & Carrion, J. Rapid ecological turnover and its impact on Neanderthal and other human populations. Trends Ecol. Evol. 22(4), 213–222 (2007).

Froehle, A. W. & Churchill, S. E. Energetic competition between Neandertals and anatomically modern humans. Palaeolanthropology 96, 119 (2009).

Harari, Y. N. Sapiens: A Brief History of Humankind (Harper Collins, New York, 2014).

Klein, R. G. The Human Career 3rd edn. (The University Of Chicago Press, Chicago, 2009).

Kolen, J. Hominids without homes: on the nature of Middle Palaeolithic settlement in Europe. The Middle Palaeolithic of Europe. In The Middle Palaeolithic Occupation of Europe (eds Roebroeks, W. & Gamble, C.) 139–175 (Leiden University Press, Leiden, 1999).

Longrich NR (2020) Did Neanderthals go to war with our ancestors? BBC Future. https://www.bbc.com/future/article/20201102-did-neanderthals-go-to-war-with-our-ancestors.

Lowe, J. et al. Volcanic ash layers illuminate the resilience of Neanderthals and early modern humans to natural hazards. Proc. Natl. Acad. Sci. 109(34), 13532 (2012).

McBrearty, S. & Brooks, A. S. The revolution that wasn't: a new interpretation of the origin of modern human behavior. J. Hum. Evol. 39, 453–563 (2000).

Melchionna, M. et al. Fragmentation of Neanderthals' pre-extinction distribution by climate change. Palaeogeogr. Palaeoclimatol. Palaeoecol. 496, 146–154 (2018).

Mellars, P. Neanderthals and the modern human colonization of Europe. Nature 432, 461–465 (2004).

Mithen, S. J. Domain-specific intelligence and the Neanderthal mind. In Modelling the Early Human Mind (eds Mellars, P. & Gibson, K.) 217–229 (Oxbow Books, Oxford, 1996).

Mithen, S. J. The Prehistory of the Mind: The Cognitive Origins of Art, Religion and Science (Thames & Hudson, London, 1999).

Muller, A. & Clarkson, C. Identifying major transitions in the evolution of lithic cutting edge production rates. PLoS ONE 11(12), 0167244 (2016).

Pereira-Pedro, A. S., Bruner, E., Gunz, P. & Neubauer, S. A morphometric comparison of the parietal lobe in modern humans and Neanderthals. J. Hum. Evol. 142, 102770 (2020).

Raichlen, D. A., Armstrong, H. & Lieberman, D. E. Calcaneus length determines running economy: implications for endurance running performance in modern humans and Neandertals. J. Hum. Evol. 60(3), 299–308 (2011).

Ramirez-Rozzi, F. V. & Bermudez de Castro, J. M. Surprisingly rapid growth in Neanderthals. Nature 428, 936–939 (2004).

Richards, M. P. et al. Stable isotope evidence for increasing dietary breadth in the European mid-Upper Palaeolithic. Proc. Natl. Acad. Sci. 98(11), 6528–6532 (2001).

Roebroeks, W. & Villa, P. On the earliest evidence for habitual use of fire in Europe. Proc. Natl. Acad. Sci. 108(13), 5209–5214 (2011).

Shipman, P. The Invaders: How Humans and Their Dogs Drove Neanderthals to Extinction (Belknap Press of Harvard University Press, Cambridge, 2015).

Sorensen, A. C. On the relationship between climate and Neandertal fire use during the Last Glacial in south-west France. Quat. Int. 436, 114–128 (2017).

Sorensen, M. V. & Leonard, W. R. Neandertal energetics and foraging efficiency. J. Hum. Evol. 40, 483–495 (2001).

Staubwasser, M. et al. Impact of climate change on the transition of Neanderthals to modern humans in Europe. Proc. Natl. Acad. Sci. 115(37), 9116–9121 (2018).

Stewart, J. R. et al. Palaeoecological and genetic evidence for Neanderthal power locomotion as an adaptation to a woodland environment. Quat. Sci. Rev. 217, 310–315 (2019).

Timmermann, A. Quantifying the potential causes of Neanderthal extinction: Abrupt climate change versus competition and interbreeding. Quat. Sci. Rev. 238, 106331 (2020).

Traynor, S., Gurtov, A. N., Senjem, J. H. & Hawks, J. Assessing eye orbits as predictors of Neandertal group size. Am. J. Phys. Anthropol. 157(4), 680–683 (2015).

Trinkaus, E. Late Pleistocene adult mortality patterns and modern human establishment. Proc. Natl. Acad. Sci. 108(4), 1267–1271 (2011).

Underdown, S. A potential role for transmissible spongiform encephalopathies in Neanderthal extinction. Med. Hypotheses 71(1), 4–7 (2008).

Wynn, T. & Coolidge, F. L. The expert Neandertal mind. J. Hum. Evol. 46(4), 467–487 (2004).

Zilhão, J. et al. Last interglacial Iberian Neandertals as fisher–hunter–gatherers. Science 367(6485), 7943 (2020).

SI Table 2: "Other factors" responses coming out of the survey

Response	Categorization	Secondary categorization
smaller bodies are cheaper to run	COMP	
Rate of cultural innovation (due to larger pop size)	COMP	DEM
lower dietary intake; better able to survive famine	COMP	
Cognitive metaplasticity and behavioral flexibility	COMP	
language	COMP	
Broader range of ecological niches (or maybe that's just economic)	COMP	
Adaptability to new niches	COMP	
Energetics and physiological demands	COMP	
Body type more efficient for covering large distances	COMP	
versatility	COMP	
social issues	COMP	DEM
Adapted to a variety of environments	COMP	ENV
More agressivity? Higher carrying capacity due to wider range of food sources?	COMP	
Ecological plasticity	COMP	
Networks and mobility	COMP	
rapid adaptive response to climatic changes	COMP	
complex clothing	COMP	
Environmental plasticity/versatility	COMP	ENV
Advantages in communication, socially adquired	COMP	
seamed clothing	COMP	
racism	COMP	
tradition	COMP	
Adaptability	COMP	
diet breadth	COMP	
energetics, body mass, and body proportions	COMP	
Lower calroic requirement for AMH (morphological advantage?) making it easier to support more individuals.	COMP	
Cultural adaptations to new environments	COMP	
presence of other hominins	COMP	
niche construction	COMP	
Intensive orientation along river courses	COMP	
Landscape modifications	COMP	
The encounter of new population in unknown terrain	COMP	
networking and interaction between fragmented, isolated local populations	COMP	
competition with other hominids	COMP	
competition with other hominins	COMP	
cultural	COMP	
food - climate	COMP	
Genetic resistance to diseases	COMP	
out-competed by H. sapiens	COMP	
recurrent population pressure on big game resources in key regions (eg Levant)	COMP	

Securing sufficient calories	COMP	
demography	DEM	
Population size (linked to economic)	DEM	
birth intervals, mobility, family size	DEM	
demographic ones	DEM	
Social group size	DEM	
Demography	DEM	
Population size	DEM	
population size	DEM	
wider distribution e.g. Africa	DEM	
more number birth	DEM	
Increases in birth rates	DEM	
greater AMH population size and territory in Africa	DEM	
numbers	DEM	
Pop size	DEM	
Group size	DEM	COMP
Swamping of population by southern migrates in part due to Family size and birth intervals	DEM	
Child mortality (illness related)	DEM	
demographic isolation	DEM	
geographic distance	DEM	
geographic isolation	DEM	
group isolation	DEM	
group size	DEM	
Group size	DEM	
High adult mortality	DEM	
isolation of groups	DEM	
Metapopulation structure	DEM	
Mortality rates	DEM	
Population distribution	DEM	
reproductive rates and pregnancy duration	DEM	
resorbed back into main Homo sapiens population via interbreeding	DEM	
The conductivity bwtween sub populations	DEM	
disease resistance	ENV	
Geographical location of refugia during climate downturns; and difference in mobility systems	ENV	
local catastrophic events	ENV	
VULCANISM	ENV	
environmental fluctuations	ENV	
animal migrations	ENV	
volcanos eruption 40.000 ka which affected Europe	ENV	
Natural disasters	ENV	
Short and fast environmental changes could lead to competition on territories in Neanderthal populations prior to AMHs appearence in Europe	ENV	
Cold	ENV	
Changes in resource availability	ENV	
change of food	ENV	

vulcano eruption CI	ENV
Diseases	ENV
immune system/ infections	ENV
Introduced diseases	ENV
Lowering of carrying capacity, due to food shrtening (e,g, big preys)	ENV
Genetically swamped	Other
genetic dilution by AMH	Other
Timing fluctuations	Other
The environment had little to nothing to do with Neandertals disappearance	Other
luck?	Other

SI Table 3: Advantages of Neanderthals over AMHs

Response	Categorization
tougher and stronger (brawn versus brain)	COMP
Longer term occupation of Eurasia, thus greater familiarity with ecology, animal behavior, environment, climate, landscape, etc.	COMP
local knowledge	COMP
Long lasting the glacial periods	COMP
morphology, immunity and different cognition	COMP
Body type better adapter to cold	COMP
cold adapted	COMP
Physiological adaptations to northern climates	COMP
Probably able to exist better in colder climates with unpredictable resources	COMP
Strenght, some immunity	COMP
adapted phenotype, """"local"""" knowledge	COMP
Adapted to colder environments	COMP
Adaptation to overcome local constrains	COMP
Physical adaptation to Europe	COMP
Adaptation to cold environment	COMP
physiological cold adaptation	COMP
cold tolerance	COMP
Long-term adaptation to Pleistocene Europe	COMP
experience with strong seasonality	COMP
Landscape knowledge	COMP
Robusticity (physical, stamina)	COMP
adaption to local environments	COMP
Kowdledge of the territory	COMP
Adaptation to cold	COMP
knoledge of the context	COMP
local knowledge of environment/habitat; physical strength	COMP
Adaptation to cold	COMP
hundreds of thousands of years of adaptation to Eurasia	COMP
experience	COMP
longer experience in seasonal, Pleistocene landscapes	COMP
Intrinsic resistance to cold	COMP
local environment adaptations to Europe	COMP
familiarity with landscape	COMP
adatation	COMP
possibly greater propensity to cooperate, delay consumption, transport richer resources over longer distances to camp	COMP
knowledge of local environments	COMP
Local physiological adaptations.	COMP
Climatic adaptations	COMP
Different mating strategies?	DEM

Full questionnaire

(including consent form)

Neanderthals 1

Survey Flow

EmbeddedData
sValue will be set from Panel or URL.

Block: consent (3 Questions)

Branch: New Branch
If
If Consent If you consent to participate in this study, please click "I agree to participate". If yo... I do not agree to participate Is Selected

EndSurvey:

Standard: intro (1 Question)
Standard: Neanderthals Opinions (5 Questions)
Standard: Neanderthals Actions (9 Questions)
Standard: climate chnage (1 Question)
Standard: Social dominance orientation (2 Questions)
Standard: Speciesism (1 Question)
Standard: demographics (11 Questions)
Standard: End (1 Question)

Page Break

Start of Block: consent

repeat
This study aims to assess the status of the ongoing debate about the replacement of Neanderthals by Anatomically Modern Humans, and to assess palaeolithic archaeologists' opinions about a couple of other, societally relevant issues. It is being conducted by Gerrit Dusseldorp (Leiden University, Faculty of Archaelogy), Krist Vaesen (Leiden University, Faculty of Archaelogy), and Mark Brandt (Tilburg University, School of Social and Behavioral Sciences).

We have advertised this study in multiple locations.

Please only take it once. Thank you!

If you have not taken the study before, please click the button below to start the study.

Page Break

consent1
Study Title: Attitudes and Opinions about Neanderthals and Anatomically Modern Humans **Consent to Participate in Research** **Description** This is a survey study. In the study we are interested in your opinion on the replacement of Neanderthals by Anatomically Modern Humans. Further, we would like to know your opinion on a couple of other issues relevant to yourself and society. You will also be asked to answer some demographic questions. It will take 5-10 minutes of your time. **Benefits** This study will contribute to Palaeolithic archaeology's disciplinary self-understanding. Accordingly, as a researcher within Palaeolithic archaeology (broadly conceived), you may benefit from the results of this study. You will not be paid for your participation. Still, at the end of the questionnaire, you may decide to sign up for a lottery and stand a chance to win a $500 gift certificate for an ancient DNA test or an Amazon voucher. **Risks/Discomforts** There is a very small risk that, given the nature of the questions you will be asked, you may become uncomfortable. If at any point during the study proceedings you become uncomfortable, you may elect to withdraw your consent to participate without consequences to you. To withdraw your consent, simply close this browser window. If any feelings of discomfort emerge, you are free to contact the lead investigator with any questions or concerns using the email address listed at the bottom of this consent form. **Confidentiality** All of the information that we obtain from your session is confidential. We will NOT collect any personal data from which you could be identified (e.g., your name, address, email etc). The data we collect for this study may be made available to researchers via accessible data repositories and possibly used for novel academic purposes. The anonymized data will be stored for at least 10 years.
If you choose to enter the lottery, we will ask for your email address, this email address will be stored separately and will not be linked in any way to the contents of the survey, will never be shared, and will be destroyed as soon as the lottery is finished. **Voluntary Nature of Participation** Your decision to participate in this study is completely voluntary. Thus, you may refuse to join the study or terminate your participation at any time without any consequences. Should you wish to terminate your participation at any point, you only need to close the browser window. **Contact Information** If you have any questions or concerns about this study, you may contact the lead investigator of this study: Gerrit Dusseldorp at g.l.dusseldorp@arch.leidenuniv.nl. The study was approved by the Ethics Committee of the Faculty of Humanities and Faculty of Archaeology of Leiden University. If you have any remarks or complaints regarding this research, you may also contact the Ethics Committee via ethics@hum.leidenuniv.nl.

---

consent2 **Consent**If you consent to participate in this study, please click "I agree to participate". If you do not consent to participate, please close the browser window.

○ I agree to participate (1)

○ I do not agree to participate (2)

End of Block: consent

Start of Block: intro

intro Thank you for participating in our study! We appreciate your time.
In this study we are interested in your opinion on the disappearance of the Neanderthal phenotype. Further, we would like to know your opinion on a couple of other issues relevant to yourself and society.
On the next 6 pages, you will complete short measures asking about these issues, as well as questions related to your demographics and background.

End of Block: intro

Start of Block: Neanderthals Opinions

neaderintro We would like to know your opinion on the disappearance of the Neanderthal phenotype.
Below you will find a list of factors that might have contributed to this disappearance.
For each factor, please provide your estimate of the strength of the factor's contribution. (0 - no contribution; 6 - strong contribution; don't know)

demo *Demographic factors pertaining to Neanderthal populations*

	0 no contribution (1)	1 (2)	2 (3)	3 (4)	4 (5)	5 (6)	6 strong contribution (7)	don't know (8)
Inbreeding (1)	○	○	○	○	○	○	○	○
Stochasticity (random fluctuations in birth and mortality rates) (2)	○	○	○	○	○	○	○	○
Population size (3)	○	○	○	○	○	○	○	○
Allee effects (problems of mate-finding) (4)	○	○	○	○	○	○	○	○
Other demographic factors	○	○	○	○	○	○	○	○

(which ones?) (5)	

comp *Factors pertaining to (in)direct competition with Anatomically Modern Humans (AMHs)*

	0 no contribution (1)	1 (2)	2 (3)	3 (4)	4 (5)	5 (6)	6 strong contribution (7)	don't know (8)
Cognitive advantage(s) of AMHs over Neanderthals (1)	○	○	○	○	○	○	○	○
Technological advantage(s) of AMHs over Neanderthals (2)	○	○	○	○	○	○	○	○
Social advantage(s) of AMHs over Neanderthals (3)	○	○	○	○	○	○	○	○
Economic advantage(s) of AMHs over Neanderthals (4)	○	○	○	○	○	○	○	○
Morphological advantage(s) of AMHs over Neanderthals (5)	○	○	○	○	○	○	○	○
Other advantage(s) of AMHs over Neanderthals (which ones?) (6)	○	○	○	○	○	○	○	○
Advantages of Neanderthals over AMHs (which ones?) (7)	○	○	○	○	○	○	○	○

environ *Environmental factors affecting Neanderthal populations*

	0 no contribution (1)	1 (2)	2 (3)	3 (4)	4 (5)	5 (6)	6 strong contribution (7)	don't know (8)
Climatic factors (1)	○	○	○	○	○	○	○	○
Epidemics (2)	○	○	○	○	○	○	○	○
Other environmental factors (which ones?) (3)	○	○	○	○	○	○	○	○

other Please indicate any other factor (not mentioned above) that you think contributed to the disappearance of the Neanderthal phenotype.

____

____

____

____

____

End of Block: Neanderthals Opinions

Start of Block: Neanderthals Actions

actionintro Below you will find the same list of factors that might have contributed to the disappearance of the Neanderthal phenotype.
For each factor, please check the factors that you have subscribed to in your own work over the last 5 years. **Check as many as apply**. If you have not subscribed to any of the factors, please check the box “I have not subscribed to any of these factors

actdem *Demographic factors pertaining to Neanderthal populations (check as many as apply)*

- ☐ Inbreeding (1)
- ☐ Stochasticity (2)
- ☐ Population size (3)
- ☐ Allee effects (4)
- ☐ Other demographic factors (5)

---

actcomp *Factors pertaining to (in)direct competition with Anatomically Modern Humans (AMHs) (check as many as apply)*

- ☐ Cognitive advantage(s) of AMHs over Neanderthals (1)
- ☐ Technological advantage(s) of AMHs over Neanderthals (2)
- ☐ Social advantage(s) of AMHs over Neanderthals (3)
- ☐ Economic advantage(s) of AMHs over Neanderthals (4)
- ☐ Morphological advantage(s) of AMHs over Neanderthals (5)
- ☐ Other advantage(s) of AMHs over Neanderthals (6)
- ☐ Advantages of Neanderthals over AMHs (7)

---

actenviron *Environmental factors affecting Neanderthal populations (check as many as apply)*

- ▢ Climatic factors (1)
- ▢ Epidemics (2)
- ▢ Other environmental factors (3)

---

actnone

- ▢ I have not subscribed to any of these factors (1)

---

actother Are there any other factors that you have subscribed to? Which ones?

____

____

____

____

____

Page Break

certain In the above, you have indicated the factors that you think best explain the disappearance of the Neanderthal phenotype. Please estimate your certainty regarding your explanation(s).

- ○ very uncertain1 (1)
- ○ 2 (2)
- ○ 3 (3)
- ○ 4 (4)
- ○ 5 (5)
- ○ 6 (6)
- ○ very certain7 (7)

---

metaphor On a more general note, which metaphor do you think best captures the overall structure of the development of the hominin family?

- ○ Tree (1)
- ○ Bush (2)
- ○ Braided Stream (3)

Page Break

midtext
Thank you for your responses so far. We have just 3 short pages of questions left, plus a page containing questions about demographic information.

On the next three pages, we turn our focus to some factors affecting contemporary society. We are trying to learn more about how you see society and yourself.

End of Block: Neanderthals Actions

Start of Block: climate chnage

climate Please indicate the extent to which you agree or disagree with each of the following items about the effects of climate change. (1 = strongly disagree, 7 = strongly agree)

	1 Strongly disagree (1)	2 (2)	3 (3)	4 (4)	5 (5)	6 (6)	7 Strongly agree (7)	don't know (8)	Prefer not to respond (9)
MY local area is likely to be affected by climate change (1)	o	o	o	o	o	o	o	o	o
Climate change will mostly affect areas that are far away from here (2)	o	o	o	o	o	o	o	o	o
Climate change will mostly affect developing countries (3)	o	o	o	o	o	o	o	o	o
Climate change is likely to have a big impact on people like me (4)	o	o	o	o	o	o	o	o	o

End of Block: climate chnage

Start of Block: Social dominance orientation

agree Describe yourself as you generally are now, not as you wish to be in the future. Describe yourself as you honestly see yourself, in relation to other people you know of the same sex as you are, and roughly your same age. Indicate for each statement whether it is accurate or inaccurate as a description of you. (1 = Very Inaccurate, 5 = Very Accurate)

	1 Very Inaccurate (1)	2 Moderately Inaccurate (2)	3 Neither Accurate Nor Inaccurate (3)	4 Moderately Accurate (4)	5 Very Accurate (5)	don't know (6)	Prefer not to respond (7)
I sympathize with others' feelings. (1)	o	o	o	o	o	o	o
I feel others' emotions. (2)	o	o	o	o	o	o	o
I am not really interested in others. (3)	o	o	o	o	o	o	o
I am not interested in other people's problems. (4)	o	o	o	o	o	o	o

sdo Below are several ideas about society that people may support or oppose. Show how much you favor or oppose each idea below by selecting a number from 1 to 7 on the scale below. You can work quickly; your first feeling is generally the best.

	1 Strongly disagree (1)	2 (2)	3 (3)	4 (4)	5 (5)	6 (6)	7 Strongly agree (7)	don't know (8)	Prefer not to respond (9)
An ideal society requires	o	o	o	o	o	o	o	o	o

some groups to be on top and others to be on the bottom (1)									
No one group should dominate in society. (2)	○	○	○	○	○	○	○	○	○
Group equality should not be our primary goal. (3)	○	○	○	○	○	○	○	○	○
We should work to give all groups an equal chance to succeed. (4)	○	○	○	○	○	○	○	○	○

End of Block: Social dominance orientation

Start of Block: Speciesism

spec We would you like to know how you think about issues related to nonhuman animals. Please answer the following questions based on your own opinion. (1 = strongly disagree, 7 = strongly agree)

	1 Strongly disagree (1)	2 (2)	3 (3)	4 (4)	5 (5)	6 (6)	7 Strongly agree (7)	don't know (8)	Prefer not to respond (9)
Morally, animals always count for less than humans. (1)	○	○	○	○	○	○	○	○	○

Humans have the right to use animals however they want to. (2)	○	○	○	○	○	○	○	○	○
It is morally acceptable to keep animals in circuses for human entertainment. (3)	○	○	○	○	○	○	○	○	○
It is morally acceptable to trade animals like possessions. (4)	○	○	○	○	○	○	○	○	○
It is morally acceptable to perform medical experiments on animals that we would not perform on any human. (5)	○	○	○	○	○	○	○	○	○

End of Block: Speciesism

Start of Block: demographics

demintro Thank you for answering our questions about how you see yourself and society.
This is the last page of questions.
Please answer the following questions about your demographics and profession.

gender To which gender identity do you most identify?

- ◯ Female (1)
- ◯ Male (2)
- ◯ Not listed (3) ____
- ◯ Prefer not to answer (4)

---

*

age What is your age?

____

---

position What is your current position?

- ◯ Master student (1)
- ◯ PhD student (2)
- ◯ Postdoc or equivalent (3)
- ◯ Assistant professor (non-tenure track) or equivalent (4)
- ◯ Assistant professor (tenure track) or equivalent (5)
- ◯ Associate professor or equivalent (6)
- ◯ Full professor (7)
- ◯ Other (please specify) (8)

____

---

familiar To what extent are you familiar with recent research in Neanderthal archaeology?

- very unfamiliar1 (1)
- 2 (2)
- 3 (3)
- 4 (4)
- very familiar5 (5)

---

subfield In which subfield(s) do you work?

- Palaeoanthropology (1)
- Archaeology (2)
- Biochemistry & Palaeo-genetics (3)
- Other (please specify) (4) ____

---

period Which period(s) do you specialise in?

- ▢ Lower and Middle Palaeolithic (1)
- ▢ Lower and Middle Stone Age (2)
- ▢ Upper Palaeolithic (3)
- ▢ Later Stone Age (4)
- ▢ Other (please specify) (5) ____

---

nation What is your nationality?

▼ Afghanistan (1) ... Zimbabwe (196)

---

country What country do you currently work in?

▼ Afghanistan (1) ... Zimbabwe (196)

---

phdcountry What country did you receive your PhD in? If you did not receive your PhD yet, which country will you likely receive your PhD in?

▼ Afghanistan (1) ... Zimbabwe (196)

---

phdlength How long have you had your PhD?

▼ Do not have a PhD (1) ... 50 years or more (52)

End of Block: demographics

Start of Block: End

end Thank you for completing our study. Your responses have helped us learn more about how researchers think about Neanderthals and AMHs. We appreciate your help!

If you have any questions or concerns about this study, you may contact the lead investigator of this study: Gerrit Dusseldorp at g.l.dusseldorp@arch.leidenuniv.nl.

By clicking the button below, you will save your data. Then, you will be redirected to a new page where you may provide your email to enter the lottery for the $500 gift certificate.

End of Block: End
